# Stepping into a dangerous quagmire: Macroecological determinants of *Bothrops* envenomings, Brazilian Amazon

**DOI:** 10.1371/journal.pone.0208532

**Published:** 2018-12-06

**Authors:** João Arthur Alcântara, Paulo Sérgio Bernarde, Jacqueline Sachett, Ageane Mota da Silva, Samara Freire Valente, Henry Maia Peixoto, Marcus Lacerda, Maria Regina Oliveira, Ivan Saraiva, Vanderson de Souza Sampaio, Wuelton Marcelo Monteiro

**Affiliations:** 1 Escola Superior de Ciências da Saúde, Universidade do Estado do Amazonas, Manaus, Brazil; 2 Diretoria de Ensino e Pesquisa, Fundação de Medicina Tropical Dr. Heitor Vieira Dourado, Manaus, Brazil; 3 Laboratório de Herpetologia, Centro Multidisciplinar, Campus Floresta, Universidade Federal do Acre, Cruzeiro do Sul, AC, Brazil; 4 Diretoria de Ensino e Pesquisa, Fundação Alfredo da Matta, Manaus, Brazil; 5 Instituto Federal do Acre, Campus de Cruzeiro do Sul, Cruzeiro do Sul, Acre, Brazil; 6 Núcleo de Medicina Tropical, Universidade de Brasília, Brasília, Brazil; 7 Centro Gestor e Operacional do Sistema de Proteção da Amazônia, Ministério da Defesa, Manaus, Brazil; 8 Sala de Análise de Situação em Saúde, Fundação de Vigilância em Saúde do Amazonas, Manaus, Brazil; Universidad de Costa Rica, COSTA RICA

## Abstract

Despite significant and successful efforts in Brazil regarding snakebites in the areas of research, antivenom manufacture and quality control, training of health professionals in the diagnosis and clinical management of bites, little is known about determinants of snakebites incidence in order to further plan interventions to reduce the impact of this medical condition. Understanding the complexity of ecological interactions in a geographical region is important for prediction, prevention and control measures of snakebites. This investigation aims to describe spatial distribution and identify environmental determinants of human envenoming by lancehead pit vipers (*Bothrops* genus), in the Brazilian Amazon. Aggregated data by the municipality was used to analyze the spatial distribution of *Bothrops* bites cases and its relationship with geographic and environmental factors. Eight geo-environmental factors were included in the analysis as independent variables: (1) tree canopy loss increase; (2) area with vegetation cover; (3) area covered by water bodies; (4) altitude; (5) precipitation; (6) air relative humidity; (7) soil moisture; and (8) air temperature. Human envenoming by lancehead pit vipers (*Bothrops* genus) in the Amazon region is more incident in lowlands [Adjusted regression coefficient [ARC] -0.0007 (IC95%: -0.001; -0.0006), p<0.0001], with high preserved original vegetation cover [ARC 0.0065 (IC95%: 0.0071; 0.0060), p<0.0001], with heaviest rainfall [ARC 0.0001 (IC95%: 0.00009; 0.0001), p<0.0001] and higher air relative humidity [ARC 0.0082 (IC95%: 0.0108; 0.0056), p<0.0001]. This association is interpreted as the result of the higher prey availability and further abundance of pit vipers in such landscapes.

## Introduction

**Fig 1:**
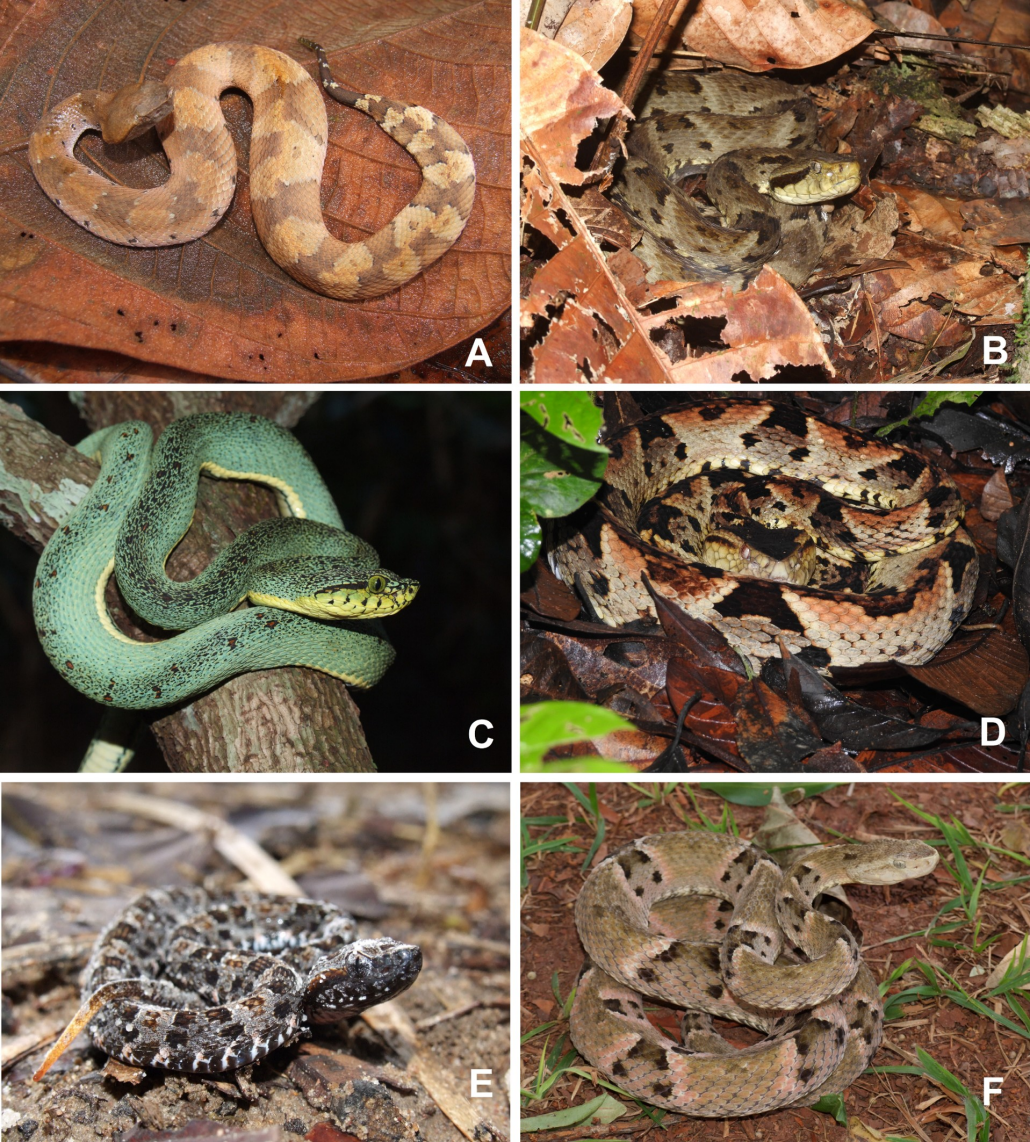
Some species responsible for *Bothrops* envenomings in the Brazilian Amazon and surrounding cerrado areas. a) *Bothrocophias hyoprora*; b) *Bothrops atrox*; c) *Bothrops bilineatus bilineatus*; d) *Bothrops brazili*; e) *Bothrops moojeni*; f) *Bothrops mattogrossensis*.

The neotropical pit viper clade of *Bothrops* and *Bothrocophias* is distributed throughout South America and associated continental islands, and includes species that range into Central America, Mexico, and the Caribbean [1–4]. Commonly known as lanceheads, the group comprises 47 species allocated in discrete species groups [4–9]. Within *Bothrops*, several species groups have been repeatedly proposed and named: *Bothrops alternatus* group, *Bothrops neuwiedi* group, *Bothrops jararaca* group, *Bothrops atrox* group and *Bothrops taeniatus* group [9]. Species of *Bothrops* occupy all main ecosystems, from rainforests to grasslands and other dry habitats [4,10]. This genus includes mostly terrestrial species (e.g. *B*. *alternatus* and *B*. *neuwiedi*), as well as many that use vegetation, from the semi-arboreal *B*. *jararaca* [11] to the almost completely arboreal *B*. *bilineatus* [12–14]. In at least some species, such as *B*. *atrox*, juveniles are also found frequently on the vegetation in contrast with the adults [15–17]. In the Brazilian Amazon and surrouding *cerrado* areas, there are 12 species of pit vipers, belonging to the *Bothrops* and *Bothrocophias* genera. Five of them (*Bothrops lutzi*, *B*. *marmoratus*, *B*. *mattogrossensis*, *B*. *moojeni*, and *B*. *pauloensis*) are present only in *cerrado* areas, while the others are characteristic of the Amazon rainforest environments (*Bothrops atrox*, *B*. *bilineatus*, *B*. *brazili*, *B*. *marajoensis*, *B*. *taeniatus*, *Bothrocophias hyoprora* e *B*. *microphthalmus*) [4,18] (Fig 1).

*Bothrops atrox*, the Amazonian lancehead, inhabits mostly forests, although it may be occasionally found in disturbed habitats around human settlements, including deforested areas (pastures and crops) and in urban environments [4,16,18–21]. This snake is the responsible for more cases and fatalities in the Amazon than any other venomous snakes, causing 80–90% of the snake envenoming in the region [22]. This species has predominantly nocturnal activity [16,17], presenting a generalist diet, preying on centipedes, fish, anurans, lizards, other snakes, birds and small mammals [16,23–28]. Despite the wide geographic distribution in the Amazon, *B*. *atrox* venoms share the same family of toxins, as PIII and PI snake venom metalloproteinase, phospholipase A2, serine proteinase, cysteine-rich secretory protein, L-amino acid oxidase and C-type lectin-like [29,30]. A PI metalloproteinase, called batroxase, isolated from *B*. *atrox* venom, has fibrinolytic, thrombolytic activities and induces weak bleeding through the digestion of the extracellular matrix components such as laminin, type IV collagen and fibronectin [31]. In general, *Bothrops atrox* envenoming shows pain, swelling, regional lymphadenopathy, ecchymosis, blistering and necrosis as the most common local clinical manifestations [32–34]. Secondary bacterial infections were observed in around 40% of the *Bothrops* snakebites [34]. Spontaneous systemic bleeding and acute renal failure are common systemic complications after *Bothrops* envenomings [32–34].

In the Brazilian Amazon, snakebites are under the influence of precipitation, likely because snakes in the Amazon exhibit increased activity during months with heaviest rainfall. Moreover, in this period, the snakes look for upland areas during flooding, which increases the likelihood of contact between humans and snakes [35,36]. The contact between snakes and human populations is often associated with extractive or agricultural activities, which can increase snakebite burden in conditions of land management or deforestation. Urban growth provides changes in the natural habitat of snakes, which can lead to a greater probability of snakebites. Predominance of snakebites in adult men living in rural areas and/or workers involved in farming, hunting, and forestry activities suggests their occupational risk. Socio-demographic variables such as indigenous status and health system performance were previously described as associated to snakebites burden in the Amazon [37]. In this region, absence of specialized centers to treat snakebites represents a considerable obstacle to the provision of quality care to patients in remote areas. Additionally, living in places with higher performance of the health system is a protective factor for severity of snakebite. In low income regions, inadequate health services performance is a very widespread problem [38].

Despite significant and successful efforts in Brazil regarding snakebites in the areas of toxin research, antivenom manufacture and quality control, training of health professionals in the diagnosis and clinical management of bites, little is known about determinants of snakebites incidence in order to further plan interventions to reduce the impact of this medical condition. The aim of this investigation is to describe spatial distribution and identify environmental determinants of human envenoming by lancehead pit vipers (*Bothrops* genus), in the Brazilian Amazon.

## Methods

### Ethical clearance

This study was approved by the Ethics Review Board (ERB) of the *Núcleo de Medicina Tropical* of the *University of Brasília* (approval number 1.652.440/2016).

### Study design, data source and definitions

An ecological study design was carried out including the states of Acre, Amapá, Amazonas, Mato Grosso, Pará, Rondônia, Roraima, Tocantins and Maranhão, whose ecotypes are classified in the Amazon biome. The study area occupies 5,016,136.3 km^2^, corresponding to about 59% of the Brazilian territory and has a population of more than 24 million people [39]. The entire 775 second administrative level subdivisions, called municipalities, were defined as units of analysis. The cartography also used municipalities as unit of analysis, performed with QGIS (Version 2.18.17 LTR). The dependent variable for mapping was the snakebite incidence, presented as the mean absolute number of cases per year, reported from 2010 to 2015, using municipality population as the denominator, standardizing per 100,000 inhabitants. Data on the municipal populations was obtained from the 2010 official census and the intercensus projections [40]. *Bothrops* envenomings in humans are officially reported to the Brazilian Ministry of Health. The department responsible for snakebites surveillance provided the data presented here (S1 Table). Although *Bothrops* contact with humans resulting in envenoming generally is diagnosed based on the clinico-epidemiological profile, the positive predictive value of this type of identification reaches 97.8–100% compared to immunoassay techniques using monoclonal antibodies, due to the high prevalence of this genus perpetrating envenoming [32,34]. Although only the number of cases has been used for the analyses, all database variables were checked for duplicates and completeness by two independent researchers before analysis. The general characteristics of the cases, such as gender, age, anatomical region of the injury, area of occurrence, work-related injury, schooling, ethnical background, time elapsed from the bite until medical assistance and outcome were described. The rainfall climatology was used to construct seasonality maps [41].

Aggregated data by municipality was used to analyze the spatial distribution of *Bothrops* bites cases and its relationship with geographic and environmental factors. In order to assess the role of municipal level environmental features on *Bothrops* envenoming incidence, eight geo-environmental factors were included in the analysis as independent variables: (1) tree canopy loss; (2) area with vegetation cover; (3) area covered by water bodies; (4) altitude; (5) precipitation; (6) air relative humidity; (7) soil moisture; and (8) air temperature. In addition, three variables considered representative of demography and public health quality were included as potential confounders: (1) Human Population Density; (2) Health System Performance Index–Access; and (3) Health System Performance Index–Effectiveness. Besides of being previously described as associated to the outcome, these variables were available from public databases at municipal level.

### Definitions

The variables in this study were defined as follows:

#### Tree canopy loss

Average annual deforested area in the municipalities between 2007 and 2014, which was measured by the average annual percentage of the municipal area that lost forest vegetation; estimated based on the computer assisted analysis of a series of images from Lansat, Cbers, UK-2-DMC or Resourcenet. The analysis is performed by TerraLib/TerrAmazon project. The detection of deforested area, vegetation cover and cloud area are used to estimate the total increment of deforested area as described in PRODES methodology, considering the automatically detected area plus the estimated area under cloud cover, according the *Coordenadoria Geral de Observação da Terra Programa Amazônia* (PRODES) [42];

#### Area with vegetation cover

Percent (%) of municipal area covered by vegetation in 2010, automatically detected by the image processing as described in PRODES methodology [42];

#### Area covered by water bodies

Percent (%) of municipal area covered by water bodies in 2010, automatically detected by the image processing as described in PRODES methodology [42];

#### Altitude

Measured as the lowest point within a county in meters above mean sea level using the global digital elevation model geo-processed by *Agência Nacional de Aviação Civil* [43];

#### Precipitation

Defined as the deposition of water to surface of Earth, in the form of rain, snow, ice or hail. Is measured in millimeter (mm) and one millimeter of rain corresponds to 1 liter per square meter of water on the surface. In this study, we used the accumulated value in the period evaluated. This data were compose from Unified Precipitation Project that are underway at NOAA Climate Prediction Center (CPC), every day with spatial resolution of 0.5° latitude x 0.5° longitude at surface level, for the study period [44];

#### Air relative humidity

Defined as the ratio, expressed in percent, of the amount of water vapor in a given volume of air to the amount that this volume could contain if the air were saturated. This data were compose from National Centers for Environmental National Centers for Environmental Prediction (NCEP) reanalysis, every 6 hours (0 to 18) with spatial resolution of 2.5° latitude x 2.5° longitude at surface level [45];

#### Soil moisture

Defined as the water that is maintained in the spaces between soil particles (cm^3^ water/cm^3^ soil), in other words the water is available in the upper layer of soil. This data were compose from National Centers for Environmental National Centers for Environmental Prediction (NCEP) reanalysis, every 6 hours (0 to 18) with spatial resolution of 2.5° latitude x 2.5° longitude between 0–10 cm in soil [45];

#### Temperature

Defined as a quantity of heat that exists in the air and measured in degree Celsius (°C). This data were compose from National Centers for Environmental National Centers for Environmental Prediction (NCEP) reanalysis, every 6 hours (0 to 18) with spatial resolution of 2.5° latitude x 2.5° longitude at 2 meters of the surface [45];

#### Human population density

Defined as the number of people living in the municipality divided by its area in square kilometers.

#### Health System Performance Index–Acces

Composed from 16 indicators from basic health assistance, this variable is a subcomponent of an index that reflects both the potential and really obtained access to public health system [46]. This variable was included to the study because its value in health system evaluation and the influence that the health system quality could has on the outcome information.

#### Health System Performance Index–Effectiveness

This subcomponent of the index is composed from 8 indicators and reflects the effectiveness of the public health system [46]. Since this feature also influences the capacity of the health system to promote proper attention and notification, this variable was included to the model as a confounder.

### Data analysis

Duplications were solved before data analysis. The variables were first separately assessed for association with snakebite incidence. All independent variables were entered individually into a bivariate regression model and preselected if p≤0.20. Subsequently, variance inflation factor (VIF) was estimated to verify the relationship between all preselected independent variables (check for potential collinearity), in which coefficient >10 were considered high. For this study none VIFs were higher than 10. Interactions between biologically plausible variables were examined (rain vs. temperature; area with vegetation cover vs. canopy tree loss and air relative humidity vs. precipitation), if found significant (p<0.05), interaction terms were kept for further analysis. The eight geo-environmental factors were included in the analysis as independent continuous variables. A Poisson model was used to estimate the regression coefficient between snakebites incidence and the geo-environmental factors. Multivariable models were built in a manual stepwise fashion starting with the forward method, where each remaining variable was added to the best previous model, selected by the Akaike Information Criterion (AIC); in the case the variable remained numerically the same, the Bayesian Information Criterion (BIC) was used. Lastly, a backward elimination step was performed, resulting in a final model in which only variables with p <0.05 were kept. The adjustment of the final model was assessed using Pearson goodness-of-fit test, p>0.05. In addition, an adjusted model was constructed by including three variables as potential confounders into the model, with equal weights: (1) Human Population Density by municipality, assuming that snakebites are density-dependent, i.e., the contact rate between human and *Bothrops* individuals depends upon the local population density [47]; (2) Access to Health System and (3) Health System Effectiveness, subcomponents of the Mean Health System Performance Index (MHSPI) [46]. Statistical analyses were performed using the STATA statistical package version 13 (Stata Corp. 2013).

## Results

### Exploratory and descriptive analysis

According to the official reporting system, 70,816 snakebites were recorded in the Amazon Region during the study period. From this total, 13,442 cases (19.0%) were not included in the analysis because their classification as non-venomous bites (4,886 cases), *Lachesis* bites (5,217 cases), *Crotalus* bites (3,103 cases) or *Micrurus* bites (236 cases).

The total of cases eligible in this study was 57,374 *Bothrops* snakebites, resulting in an incidence rate of 37.2 cases per 100,000 inhabitants/year. There was a slight variation in the annual incidence rates during the study period. Incidence was higher in 2011 (38.8 per 100,000 inhabitants), and lower in 2015 (36.2 per 100,000 inhabitants). All the variables retrieved from the original dataset presented completeness higher than 70% (S2 Table). Most of the snakebites occurred in young males (45,091 cases; 78.6%). Regarding the area of occurrence, 86.6% were reported in rural areas. The most affected age group was between 18 and 45 years old (31,568 cases; 55.0%). Admixed population was the most reported in the ethnicity field (40,499 cases; 74.4%). The most affected education group was the group with ≤4 years of schooling (18,276 cases; 44.1%). A proportion of 40.1% of the snakebites were related to work activities. Most of the snakebites occurred in the lower limbs (83.9%). Regarding time elapsed from the bite until medical assistance, 78.3% of the cases received treatment within the first six hours after the snakebite, 16.4% within 6–24 hours and 5.3% with more than 24 hours after bite.

**Fig 2:**
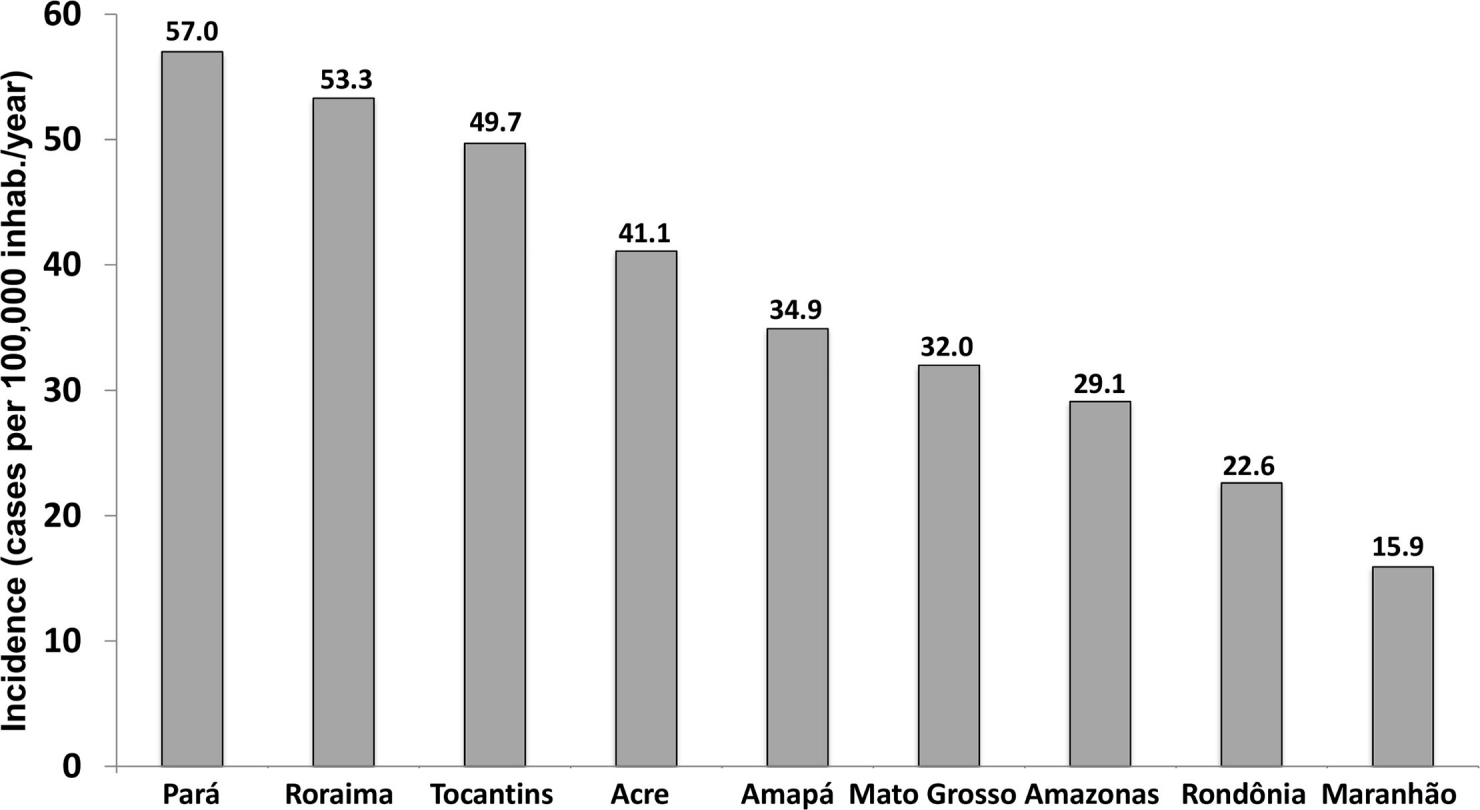
Incidence rates of *Bothrops* envenomings presented by state. The highest incidence rates were reported in the states of Pará (57.0 per 100,000 inhabitants/year), Roraima (53.3 per 100,000 inhabitants/year) and Tocantins (49.7 per 100,000 inhabitants/year). The lowest incidence rates were reported in the states of Maranhão (15.9 per 100,000 inhabitants/year), Rondônia (22.6 per 100,000 inhabitants/year) and Amazonas (29.1 per 100,000 inhabitants/year).

**Fig 3:**
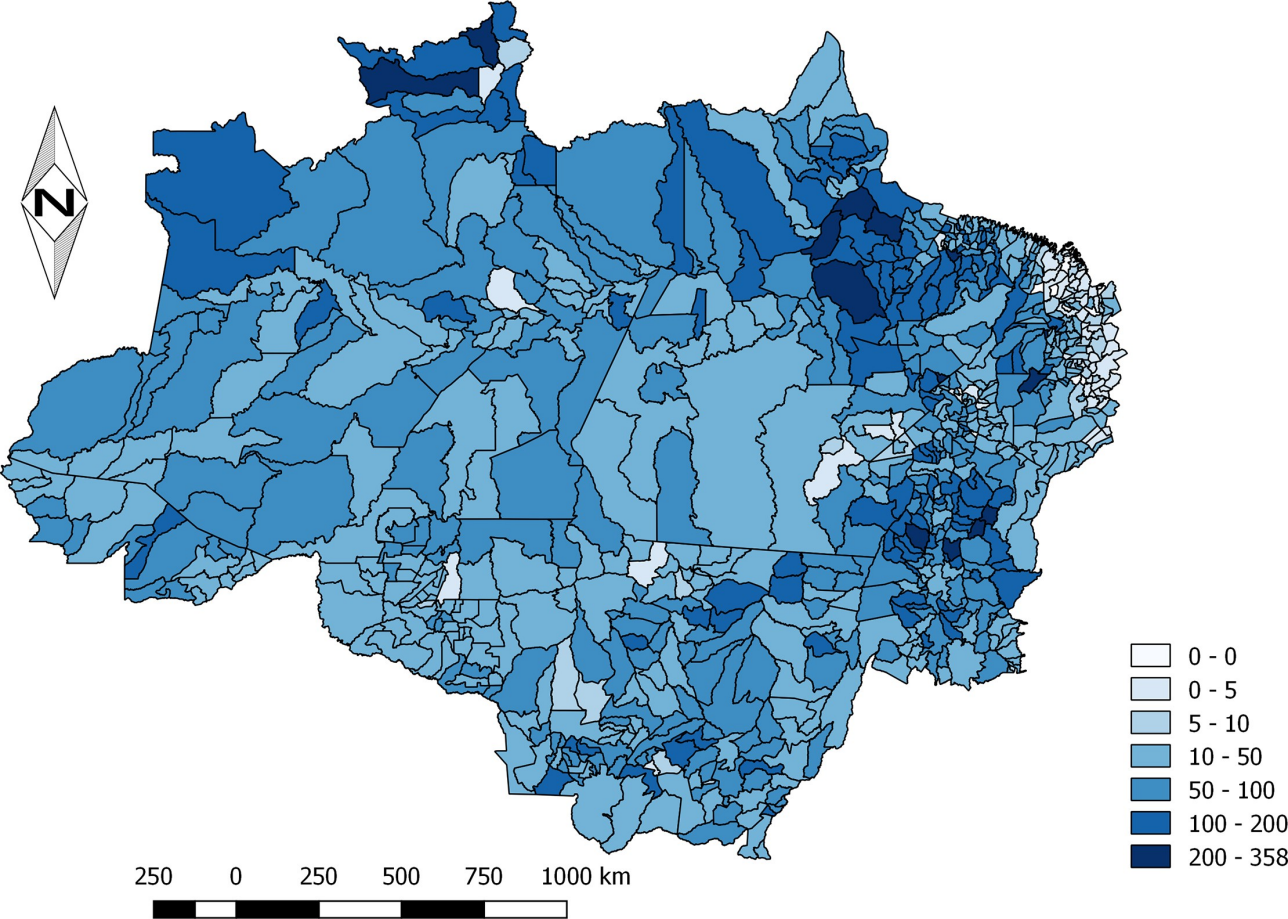
Spatial distribution of *Bothrops* snakebites in the Brazilian Amazon from 2010 to 2015. Map were created using incidence per 100,000 inhabitants. Snakebites are largely distributed in the Amazonian states, with several counties presenting incidences higher than 100 cases per 100,000 inhabitants. The Northwest of the State of Amazonas, North of Roraima, North of Pará on the border with Amapá and central part of the state of Tocantins, show spots of higher incidence rates.

Incidence rates by states are presented in Fig 2. Several municipalities had incidence rates higher than 100 cases per 100,000 inhabitants/year, distributed unevenly in the study area. The Northwest of the State of Amazonas, North of Roraima, North of Pará on the border with Amapá and central part of the state of Tocantins, show spots of higher incidence rates. The municipality of Alto Alegre, in the State of Roraima, presented the highest rate among all municipalities (358.3 per 100,000 inhabitants/year), followed by the municipalities of Anajás (338.9 per 100,000 inhabitants/year) and Afuá (303.7 per 100,000 inhabitants/year), in the State of Pará (Fig 3).

**Fig 4:**
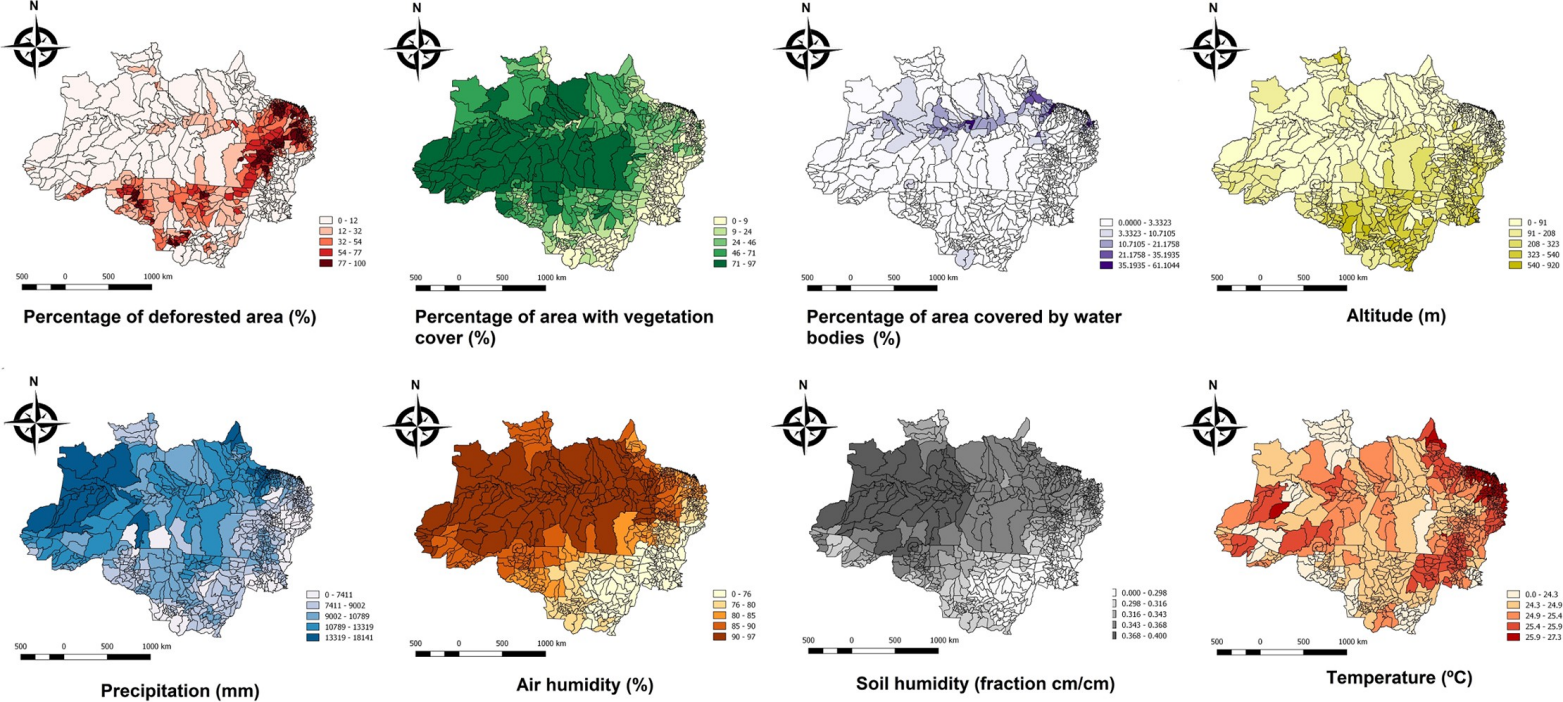
Descriptive analysis of geo-environmental variables. A) Tree canopy loss. Mean tree canopy loss in the study area was 35.6% (±33.4%), being lower in Western Brazilian Amazon, namely in the North and West of the state of Amazonas, West of the state of Acre and Amapá. A higher tree canopy loss is observed in the state of Maranhão, in the extreme East of the states of Pará and Acre, and in some municipalities of the state of Mato Grosso and Rondônia. B) Area with vegetation cover. The mean area with vegetation cover is 25.31% (±27.4%), being lower in the states of Tocantins, Maranhão and in the South of the state of Mato Grosso. C) Area covered by water bodies. The mean area covered by water bodies is 2.9% (±6.9%) and is larger in the municipalities located in the banks of the Solimões, Negro and Amazonas rivers and in the Pantanal region of the state of Mato Grosso. D) Altitude. The whole study area had a mean of 153.7 meters above sea level (±148.6 meters). Altitude is lower in municipalities located in the banks of the Amazonas river and higher in the states of Mato Grosso, Tocantins, south of the Maranhão, peaking in the north of the state of Roraima. E) Precipitation. The average value of accumulated rainfall in the study area is 9,356.2 millimeters (±2,335.1 millimeter), ranging from <5,000 millimeters in some municipalities in the periphery of the region to >13,000 millimeters in the extreme Western Amazon. F) Air relative humidity. Mean air relative humidity in the study area is 82.9% (±6.7%), ranging from ~70% in municipalities Southern Amazon border to >95% in the Western Amazon. G) Soil Moisture. Mean soil moisture in the study area is 0.3270047 cm^3^ water/cm^3^ soil (±0.0280811 cm^3^/cm^3^), being lower in the states of Maranhão, Tocantins and Mato Grosso and higher in the state of Amazonas. H) Air temperature. Mean air temperature in the region is 25.2°C (±0.81°C), ranging from ~23°C in the state of Roraima to >27°C in the extreme Eastern Amazon.

Descriptive analysis of geo-environmental variables is presented in the Fig 4.

### Univariate analysis and correlation test

Tree canopy loss and air temperature were variables negatively related to envenoming rate. Percentage of vegetation cover and precipitation were variables positively related to pit vipers snakebite incidence. Percentage of water bodies cover, altitude, air relative humidity and soil moisture were not significantly related to snakebite incidence rates in the univariate analysis (Table 1).

**Table 1:** Association of geo-environmental variables with *Bothrops* snakebite incidence rates in the Brazilian Amazon in the univariate analysis.

Variable	Mean	Crude regression coefficient [CRC] (CI 95%)	p
**Geo-environmental variables**			
Tree canopy loss (%)	35.6±33.4	**-0.0042 (-0.0062; -0.0022)**	**<0.0001**
Area with vegetation cover (%)	25.3±27.37	**0.0022 (0.0004; 0.0041)**	**0.018**
Area covered by water bodies (%)	2.9±6.9	-0.0003 (-0.0115; 0.0108)	0.953
Altitude (meters above sea)	153.7±148.6	-0.0003 (-0.0008; 0.0001)	0.181
Precipitation (millimeters)	9356.2±2335.1	**0.0001 (0.0005; 0.00009)**	**<0.0001**
Air relative humidity (%)	82.9±6.7	0.0085 (-0.0007; 0.0177)	0.071
Soil moisture (cm^3^ water/cm^3^ soil)	0.3±0.3	0.0036 (-0.0176; 0.0249)	0.736
Air temperature (°C)	25.2±0.8	**0.1102 (0.1919; 0.0284)**	**0.008**
**Adjusting variables**			
Population density	24.8±129.9	**-0.0150 (-0.0202; -0.0098)**	**<0.0001**
Access to health service	3.9±1.0	**-0.1064 (-0.1770; -0.0357)**	**0.003**
Effectiveness of health service	7.2±0.9	**0.2013 (0.1436; 0.2591)**	**<0.0001**

Human population densities, access to health system and health system effectiveness were tested in a univariate model to assess their role as potential confounders. Population density and access to health service were negatively related to snakebites incidence rate. Health system effectiveness was positively related to snakebites incidence rate.

**Fig 5:**
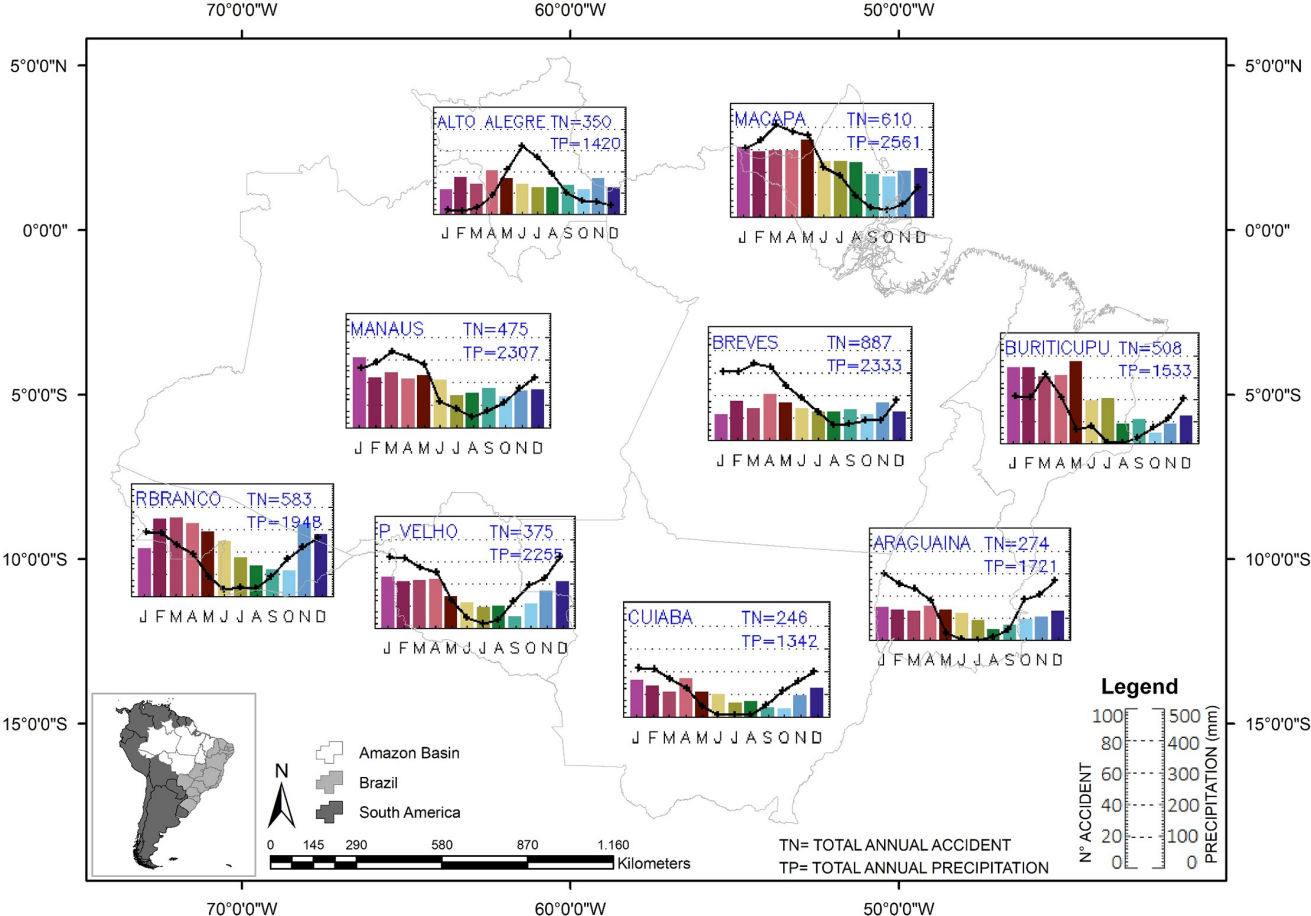
Temporal and spatial distribution of *Bothrops* envenomings and precipitation. Map was created using the absolute number of *Bothrops* snakebites and the accumulation of precipitation per month during the years studied. Seasonality of *Bothrops* bites over the year, with a pronounced increase of cases in the rainiest trimester across the study area. In the bar graph is the absolute number of snakebites and in the line graph is the cumulative value of precipitation.

There was a notable seasonality of *Bothrops* bites over the year, with a pronounced increase of cases in the rainiest trimester across the study area (Fig 5).

### Multivariable analysis

After adjustment by human population density, access to health system and health system effectiveness, altitude and air temperature were negatively related to pit vipers envenoming rate. Moreover, percentage of vegetation cover, precipitation and air relative humidity were variables positively related to *Bothrops* bite incidence (Table 2).

**Table 2:** Association of geo-environmental variables with *Bothrops* snakebite incidence rates in the Brazilian Amazon in the multivariate analysis.

Variable	Adjusted regression coefficient [ARC] (CI 95%)	p
Geo-environmental variables		
Tree canopy loss (%)	**0.0001 (-0.0002; 0.0004)**	**0.549**
Area with vegetation cover (%)	**0.0065 (0.0071; 0.0060)**	**<0.0001**
Altitude (meters above sea)	**-0.0007 (-0.001; -0.0006)**	**<0.0001**
Precipitation (millimeters)	**0.0001 (0.00009; 0.0001)**	**<0.0001**
Air relative humidity (%)	**0.0082 (0.0108; 0.0056)**	**<0.0001**
Air temperature (°C)	**-0.1605 (-0.1759; -0.1450)**	**<0.0001**

Adjusted by human population density, access to health system and health system effectiveness.

## Discussion

In the Brazilian Amazon, although urban population predominates, most of the snakebites were recorded in adult males living in rural areas. This epidemiological profile was reported previously in the Amazon [35,36], and probably is related to a significant higher exposition of this population group when exerting agricultural or forestry activities, in the places mostly inhabited by lancehead pit vipers. Several studies have reported on habitat use by *B*. *atrox*, the easiest snake to find in comparison to other species in Central Amazonia [48], and concluded that the species is mainly found on the ground or climb into understory vegetation, in the case of juveniles [15–17]. Some agroforestry activities developed in the Amazon are very conducive to contact of workers with snakes, such as the Brazil nut and palm tree fruits harvest, in which scouring the leaf litter for the collection of fallen fruits enables the frequent finding of *B*. *atrox*, *B*. *taeniatus* and *B*. *brazili* [47].

In this work, percentage of vegetation cover was positively related to *Bothrops* bite incidence, indicating that the rainforest environment maintenance is crucial for snake population density in levels that warrant an intense contact rate between *Bothrops* individuals and humans. In this preserved environment, snakes probably find more propitious microhabitats and higher prey availability [15–17,48]. Populations from a variety of taxonomic groups, including snakes and their preys are declining in response to the loss of habitats [49]. Although very less frequent, reports of urban cases are noteworthy, showing that pit vipers are able to occupy a wide environmental gradients and maintain populations in forest fragments within very anthropized areas, as observed for *B*. *atrox* in the Amazon [18], *B*. *moojeni* and *B*. *neuwiedi* in Cuiabá, Southern Amazon [50] and *B*. *jararaca* in Southern Brazil [51]. Furthermore, Amazonian cities outskirts are often encrusted in sylvatic environments, enabling snake populations to frequent the peridomiciliary areas where rodents, marsupials, frogs and other synanthropic animals serve as food sources [50]. Forestry, hunting and recreational activities are common in this urban residual forested areas or deeper in the forests along urban fringes are expect to be risk factors for snakebites [48].

In this investigation, significant higher snakebite incidence rates were found in areas with higher precipitation indexes. As previously flagged in other studies, most of the snakebites in the Amazon occur during the rainy season, with some geographical variations in the region [35,48,52,53]. Some authors suggest that this seasonal pattern is related to the flooding in areas of land adjacent to streams margins, the location of the riverine villages, forcing the snakes to upland areas, increasing the likelihood of snakebites both by increasing the density of snakes and their locomotion in the search for prey [35]. Rainfall could also mediate human-activities, such as starting the agricultural season, so increasing snakebite incidence. Moreover, it is well established that snakes in the Amazon exhibit increased activity during months with higher rainfall, including *Bothrops atrox* and *B*. *bilineatus* [13,14,16,17]. In the region of Manaus, Central Amazon, *B*. *atrox* present an unimodal seasonal activity in summer reflected in more productive collections of specimens in this season [16]. Cunha and Nascimento [19] also reported on seasonal activity in *B*. *atrox* in eastern Amazonia (decrease from June-November). Thus, the results may apply for other Amazonian localities as well and explain the notable seasonality of *Bothrops* bites over the year, with a pronounced increase of cases in the rainiest trimester across all the study area, as shown in this study. Furthermore, in the Amazon the incidence of juveniles also occurred mainly during the rainy months [13,16]. An increase in the forest productivity with a higher availability of some types of prey, such as anurans and lizards, was suggested as a cause for the higher snake abundance in the rainy season [16,17,54–56]. Rainfall, which is highly correlated with air humidity is often suggested as an important factor determining the seasonal incidence of tropical snakes [16,17,55–58], consistent with the positive relation between air relative humidity and *Bothrops* bite incidence found here.

Herein, municipalities located in the extensive lowlands of the Amazon basin presented high snakebite incidence rates. This plain area is surrounded by plateaus, located between the Guianas plateau, the Brazilian plateau and the Atlantic Ocean, in which incidence was significantly lower. Although the seasonally flooded lowland (called ‘*várzeas*’) occupies only a small part of this region, extending along the banks of the Amazon River and its tributaries, the vast expanses of low-plateaus or low-sedimentary plateaus (called ‘mainland’) are saved from common floods. However, sedimentary plateaus are the origin of numerous streams flowing into the ‘*várzeas*’ area. In this landscape, water dividers present a slightly higher altitude in relation to the parafluvial catena. These lowlands enclosed within a forest canopy are the most likely ecosystem for encountering snakes of the *Bothrops* genus [15,47]. Reptile richness and snakebites are most strongly correlated with temperature, mediated by precipitation, and decreases in high landscapes [44,59–61]. Consistently, in the Central Amazon, the distribution of *B*. *atrox* is not uniform within the forest, with a density of about 6.4 times higher near streams, probably due to the increase in prey availability [15]. As indigenous and riverside populations live chiefly in human settlements in the riverbanks, this riverscape encompasses ecological processes that conduce humans to contact snakes.

The nature of the surveillance system may have influenced record keeping. Some patients with mild bites in inaccessible areas may not be reported to hospitals and those evolving to severity may die on the way before reaching medical attention. To minimize this possible bias, environmental correlations were adjusted by *Access to Health System and Health System Effectiveness*, subcomponents of the *Mean Health System Performance Index* (MHSPI) [46]. These variables were included to adjust the possible differences in sensitivity of the surveillance case definition among municipalities across the study area, i.e., the ability of the epidemiological surveillance to identify all possible *Bothrops* snakebites in the community, depending mostly on the access of the individuals bitten to the health services and the effectiveness of this system do diagnose and report the cases properly. Considering the macroecological nature of this study, the findings presented rely on weaknesses peculiar to this type of study and further investigation must consider the role that social and economic factors related to the people affected by the snakebites might have [62]. In an ecosocial perspective, future efforts should attempt to examine interrelations between environmental, social and economic factors related to the people affected by the snakebites. Unfortunately, high-quality integrated data are still lacking for this approach in snakebite endemic areas [63].

In conclusion, human envenoming by lancehead pit vipers (*Bothrops* genus) in the Amazon region is more incident in lowlands, with high preserved original vegetation cover, with heaviest rainfall and higher air relative humidity. This association is interpreted as the result of the higher prey availability and further abundance of pit vipers in such landscapes.

## Supporting information

S1 Table Study database. (XLS)

S2 Table Epidemiological characteristics of patients who are victims of *Bothrops* snakebites, Brazilian Amazon, from 2010 to 2015. (DOCX)
