# Supplementary material for: Stepping into a dangerous quagmire: Macroecological determinants of Bothrops envenomings, Brazilian Amazon
Source: PLoS One. 2018 Dec 6;13(12):e0208532. doi: 10.1371/journal.pone.0208532 (PMC6283637; doi:10.1371/journal.pone.0208532)
Supplement: S2 Table — (DOCX) [file pone.0208532.s002.docx]

| **Characteristics (completeness)** | **Number** | **%** |
| --- | --- | --- |
| Sex (n=57,374; 100%) |  |  |
| Male | 45,091 | 78.6 |
| Female | 12,274 | 21.4 |
| Area of occurrence (n=56,666; 98.8%) |  |  |
| Rural | 49,079 | 86.6 |
| Urban/peri-urban | 7,587 | 13.4 |
| Age Group (in years) (n=57,372; ~100%) |  |  |
| 0-17 | 11,966 | 20.9 |
| 18-45 | 31,568 | 55.0 |
| 46-65 | 11,433 | 19.9 |
| ≥66 | 2,405 | 4.2 |
| Ethnicity (n=54,414; 95.7%) |  |  |
| Admixed | 40,499 | 74.4 |
| White | 5,374 | 9.9 |
| Black | 4,385 | 8.1 |
| Indian | 3,647 | 6.7 |
| Asian | 509 | 0.9 |
| Education (in years) (n=41,400; 72.2%) |  |  |
| Illiterate | 3,935 | 9.5 |
| ≤4 | 18,276 | 44.1 |
| 5-8 | 11,646 | 28.1 |
| >8 | 5,099 | 12.3 |
| Not applicable | 2,444 | 5.9 |
| Work-related accident (n=47,688; 83.1%) |  |  |
| Yes | 19,124 | 40.1 |
| No | 28,564 | 59.9 |
| Anatomical region of the bite (n=56,925; 99.2%) |  |  |
| Lower limbs | 47,764 | 83.9 |
| Upper limbs | 8,317 | 14.6 |
| Other sites | 844 | 1.5 |
| Time elapsed form bite to medical assistance (hrs) (n=54,152; 94.4%) |  |  |
| ≤6 | 42,395 | 78.3 |
| >6-24 | 8,890 | 16.4 |
| >24 | 2,867 | 5.3 |
